# Iodine prophylaxis in pregnant women in Poland - where we are? (update 2015)

**DOI:** 10.1186/s13044-015-0029-z

**Published:** 2015-12-08

**Authors:** Arkadiusz Zygmunt, Andrzej Lewinski

**Affiliations:** Department of Endocrinology and Metabolic Diseases, Medical University of Lodz, Polish Mother’s Memorial Hospital - Research Institute, Rzgowska Str. 281/289, 93-338 Lodz, Poland

**Keywords:** Iodine prophylaxis, Pregnant and breast-feeding women, Iodine deficiency

## Abstract

Introduction of the model of iodine prophylaxis based on the consumption of iodized salt in 1997 has eliminated the iodine deficiency in Poland. However, in accordance with the current recommendations, all women who are planning to be pregnant or are pregnant or breastfeeding should receive an additional dose of iodine at 150–200 mcg / day because of the increased body's need for iodine during this period. Studies show that only part of women in Poland conduct a proper iodine prophylaxis and it is often initiated in the second and third trimester of pregnancy. The authors try to analyze the possible causes of this situation, pointing out what could be done to increase the degree of compliance with iodine prophylaxis during pregnancy and physiological lactation in Poland.

## Background

Introduction of the model of iodine prophylaxis based on the consumption of iodized salt in 1997, which is still in force today, was one of the undisputed successes of health policy in Poland after the change of the economic system.

Before that, Poland, like many countries in Europe, was an example of a country lying on endemic area of a goitre of a moderate or mild degree and prevalence of goitre among schoolchildren was approximately 35 %. After the introduction of iodine prophylaxis prevalence of goitre decreased significantly and equaled approximately 1.5 %. The value of ioduria increased significantly, as well [1].

Data on the effectiveness of the conducted prophylaxis are so numerous that a wide group of doctors and the public got the message that the problem of iodine deficiency in Poland has finally been resolved. That is true, but it should be remembered that iodine intake in the population is variable and depends on many factors and it can not be ruled out that the problem of iodine deficiency will reappear in the future.

The phenomenon of re-emergence of iodine deficiency has been described, e.g. in Australia, where the introduction of iodine prophylaxis had solved the problem of iodine deficiency, but after some time the endemicity of goitre developed again [2].

Currently, the ioduria oscillates around the border values (they only slightly exceeds the threshold value considered to be sufficient), and in such situation the constraints on the consumption of salt can result in insufficient iodine intake. It is probable because Poland is taking multiple approaches to reduce the intake of salt, which is relatively large, and responsible, among others, for the development of hypertension and cardiovascular events. These actions aim at reducing salt intake in Poland to 5–6 g/day [3].

Because of this, the novel carrier of iodine are being introduced, in order to diversify the source of this element in the situation of less salt consumption. On the market there are available drinking waters either containing iodine naturally or enriched with iodine in the production process, iodine is added to animal feed, and thus milk and meat are enriched with iodine. Iodine supplementation can also be performed using complex or simple pharmacological preparations containing iodine.

### Iodine and pregnancy

Iodine prophylaxis carried out on the basis of iodization of salt in Poland - is effective so far, but it does not meet the increased demand for iodine in pregnant and breastfeeding women.

Therefore, in accordance with the recommendations of Polish Society of Endocrinology, pregnant and breastfeeding women should take an extra supplementation of iodine in dose of 150–200 mcg/day [in the form of multivitamin or simple preparation of potassium iodide (KI)] [4]. It is also suggested that such supplementation should start already in the preconceptional period [4–6] while another recommendation [7] even advocates that all women who are planning to be pregnant should take an extra supplementation of iodine. Women frequently ask how long this supplementation should precede the moment of becoming pregnant - it depends on the degree of iodine deficiency; in Polish reality, while in the general population the intake of iodine is practically correct, it should be considered that such supplementation is only meant to minimize the risk of iodine deficiency and can be used for a relatively short time, ie. 1–2 months (in cases of suspected significant iodine deficiency these indications may differ). Any excess of iodine is excreted by kidneys, so when giving the inorganic iodine in microgram dose, the risk of accumulation of iodine in the body practically does not exist.

### Compliance with recommendations of iodine prophylaxis

Even the best recommendations are effective only if they are respected. In our study, we saw that only 45 % of women takes the recommended dose of iodine; consequently ioduria in pregnant women is significantly low, indirectly proving that the supply of iodine in this group is insufficient. These data are consistent with the observations of other authors - too few pregnant women in Poland takes additional iodine supplementation [8].

We have also noticed that iodine prophylaxis is being introduced too late (in the second or third trimester of pregnancy). None of the women in our survey took iodine in the preconceptional period or in the first trimester of pregnancy.

The explanation for this situation may be the fact that many obstetricians/gynecologists starts the vitamin supplementation in advanced pregnancy, trying to ensure due supply of vitamins and minerals at the beginning of pregnancy only through proper diet. Here it is worth once again to recall that in such a situation suplementation of iodine can be carried out by giving a simple preparation of potassium iodide, remembering that it can only be obtained with prescription. In the group of women studied by us, none took KI, they took only vitamins, which suggest that this form of iodine supplementation is being ignored.

The multitude of available multivitamin, registered either as a drug or as a dietary supplement, also forces the recommendation of a preparation containing iodine. This is not only connected with the credibility of the product - as evidenced by studies, only in a small number of cases, the actual iodine content in the preparation is consistent with the amount declared by the manufacturer; most often these values differ significantly, at least +/− 20 % and more. The second issue to keep in mind is that not all the multivitamin supplements prepared for pregnant women contain iodine. So if a woman will not be indicated a proper preparation, there is a risk that at the pharmacy she will acquire a preparation that does not contain sufficient dose of iodine.

The knowledge about the consequences of a significant iodine deficiency in pregnant women is very wide. Much less information is available on the slight iodine deficiency. Capturing of such dependence is difficult due to methodological problems.

The current tools being at our disposal allow us to effectively eliminate the potential iodine deficiency in pregnant women and, therefore, eliminate all the consequences of iodine deficiency, even of a slight degree. We should implement the conclusions of the present studies into everyday medical practice.

## Conclusions

The existing model of iodine prophylaxis in Poland has proved its efficiency and has eliminated endemicity of goitre in Poland.

Supply of iodine varies with time and in the future, due to the tendency to limit salt intake, it may decrease again and become insufficient. The needs of women during pregnancy and physiological lactation are so great that the amount of iodine supplied by a common model of iodine prophylaxis is insufficient; these women should receive additional iodine supplementation at a dose of 150–200 mcg / day. Only part of the pregnant women in Poland receive proper iodine supplementation and it rarely begins during the preconceptional period or in early pregnancy. In order to ensure appropriate dose of iodine, one should carefully, depending on individual needs, select a iodine-containing preparation.
